# Analysis of the gluteus medius muscle atrophy and fat infiltration in young adults after femoral neck fracture surgery: A retrospective volumetric and composition computed tomographic analysis

**DOI:** 10.1097/MD.0000000000044490

**Published:** 2025-09-12

**Authors:** Jeong Seok Choi, SungJin Ahn, Wonseok Choi, Seonghyun Kang, Yun Ki Ryu, William T. Kent, Jong-Keon Oh, Jae-Woo Cho

**Affiliations:** a Department of Orthopaedic Surgery, Korea University Guro Hospital, Seoul, Republic of Korea; b Department of Orthopaedic Surgery, Armed Forces Capital Hospital, Seongnam, Republic of Korea; c Department of Orthopaedic Surgery, University of California, San Diego, CA.

**Keywords:** fat infiltration, femoral neck fracture, gluteus medius, muscle atrophy, three-dimensional CT reconstruction

## Abstract

Gluteus medius (GMED) muscle atrophy after femoral neck fracture surgery is common and can contribute to gait abnormalities. This retrospective study aimed to evaluate the changes in muscle volume and intramuscular adipose tissue following osteosynthesis. This retrospective study included 33 patients who underwent osteosynthesis for femoral neck fracture between April 2010 and April 2022. Bilateral hip computed tomography scans were analyzed at 5 time points: preoperative, immediate postoperative, early postoperative, intermediate postoperative, and late postoperative. Three-dimensional volumetric and muscle-to-adipose tissue ratio analyses were performed. The GMED muscle volume on the fractured side decreased by up to 20% and remained reduced by 10% even 1 year after surgery, with a 1.6 times higher muscle-to-adipose tissue ratio than that on the uninjured side. These findings suggest that GMED atrophy and compositional changes persist for more than a year, even in young healthy patients, potentially affecting their long-term gait and hip function.

## 1. Introduction

Hip abductor muscles, particularly the gluteus medius (GMED), are crucial for hip function.^[1]^ The GMED generates abduction forces at the hip joint and stabilizes the pelvis during single-leg stance.^[2]^ Hence, impairment of the GMED leads to hip instability and pelvic postural imbalance during walking, resulting in the Trendelenburg sign.^[3]^ The recovery of hip abductor muscle strength is crucial after a hip fracture, and inadequate rehabilitation of these muscles can lead to an increased risk of falls and delayed recovery.^[4]^ Therefore, optimizing hip abductor strength during postoperative rehabilitation is essential not only for restoring normal gait but also for preventing long-term functional impairment and improving the overall quality of life of patients recovering from hip fractures.^[5]^

Femoral neck fractures are commonly reported in the literature as fractures associated with significant functional gait abnormalities.^[6]^ This is because femoral neck fractures directly affect the hip abductor moment arm following osteosynthesis, through mechanisms such as shortening and angulation.^[7]^ Indirectly, these fractures predispose the abductor muscles to muscle atrophy because of disuse.^[8]^ In young patients, the functional outcomes of osteosynthesis following femoral neck fractures are not always promising,^[9]^ highlighting the importance of not only surgical improvements aimed at reducing shortening and preventing nonunion and avascular necrosis but also postoperative rehabilitation strategies focused on restoring and strengthening the surrounding muscles. This remains a challenging fracture, with substantial potential for clinical improvement. Although research has been conducted on abductor muscle atrophy and fall-related proximal femoral fractures in the older population, research on changes in abductor muscle atrophy following femoral neck fracture surgery is limited.^[10]^

After femoral neck fracture surgery, the surrounding muscles atrophy. Several studies have used computed tomography (CT)-based analysis to assess muscle volume and muscle-to-adipose tissue (MAT) ratio. Miller et al reported that 2 months after hip fracture repair, older adults showed muscle atrophy with increased intermuscular fat and decreased muscle attenuation in the fractured limb based on mid-thigh cross-sectional area (CSA) data.^[11]^ Ay et al found similar atrophy at the surgical site following total hip arthroplasty using the CSA data of the psoas and paravertebral muscles.^[12]^

CSA values are often used as representative markers of muscle volume; however, such measurements in cross-sectional images are widely variable and depend on the location of the section.^[3]^ Considering the unique anatomical characteristics of the GMED, which originates from the gluteal surface of the ilium and inserts into the greater trochanter of the femur in a fan-shaped manner, three-dimensional (3D) analysis can be more accurate than CSA studies, and we decided to apply this approach to the GMED. Therefore, this study aimed to investigate the 3D volume and MAT changes in the GMED after osteosynthesis in femoral neck fractures in young patients using CT-based analysis.

## 2. Methods

### 2.1. Patients

This retrospective study was conducted at a single university hospital and was approved by the Institutional Review Board of our hospital. Patients aged <65 years with femoral neck fractures who underwent osteosynthesis between April 2010 and April 2022 were enrolled in the study. To evaluate changes in the gluteus muscle volume over time after surgery, this study focused on patients who underwent a minimum of 3 or more CT scans with a region of interest encompassing the entire bilateral GMED. Patients with bilaterally operated hip, previous hip surgery, neuromuscular disease, pathologic fracture, and/or inability to undergo rehabilitation therapy for an extended period because of severe trauma-induced injuries were excluded. Finally, 33 patients were enrolled in this study.

Bilateral hip CT scans were obtained before and after surgery. Each CT scan was categorized into 5 groups based on the time of acquisition: preoperative, immediate postoperative, early postoperative (up to 4 months after surgery), intermediate postoperative (5–11 months after surgery), and late postoperative (>12 months after surgery) (Table 1). According to this classification, the mean values of the GMED volume and MAT ratio were calculated for each group, and statistical comparisons were performed between the fractured and uninjured sides.

**Table 1:** Definitions of each time group and the corresponding number of patients.

Group	Description	Numbers
Preoperative	Number of patients before the surgery	33
Immediate postoperative	Number of patients immediately after the surgery	32
Early postoperative	Number of patients up to 4 mo after surgery	18
Intermediate postoperative	Number of patients from 5 to 11 mo after surgery	17
Late postoperative	Number of patients at ≥12 mo after surgery	16

### 2.2. Rehabilitation program

Immediate hip range of motion and isokinetic hip abductor strengthening exercises were initiated postoperatively. Non-weight-bearing crutch gait was maintained for the first 4 weeks postoperatively, followed by a transition to partial weight-bearing. Weight-bearing gradually increased, and at 3 months post-surgery, full weight-bearing was achieved without the need for crutches. The entire process was performed under the supervision of an orthopedic trauma surgeon.

### 2.3. Imaging study

Following admission to the emergency room, pelvic CT was performed in all patients within 24 hours of injury. The imaging procedure used a multi-slice, multi-detector helical scanner. Although diverse protocols were used based on the clinical examinations conducted, standard CT examination consistently included the region from the iliac crest to one-third of the proximal femur to encompass the entire GMED. The acquired images were assessed at an axial slice thickness of 2 mm, and additional coronal and sagittal reconstructions were performed for precise measurements.

**Figure 1.**
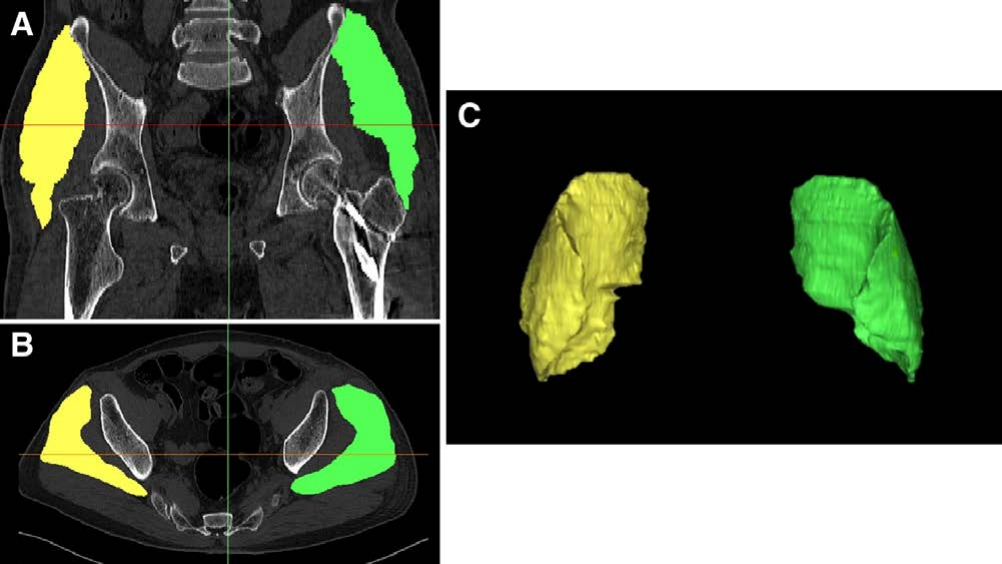
(A) Coronal computed tomography (CT) slice of both hips. Yellow, gluteus medius (GMED) of the uninjured side; green, GMED of the fractured side after surgery. (B) Axial CT slice from the same patient. (C) Three-dimensional reconstruction of bilateral GMED muscles.

Muscle volume was measured using a 3D medical image-processing software (Materialise Mimics; Materialise NV, Leuven, Belgium). This software provides functionalities for quantifying distances, areas, volumes, and radiological density. The 3D muscle volume of the GMED was measured using a freehand drawn approach, wherein the contours of the GMED were delineated and tracked for accurate measurements. The entire muscle was meticulously identified by excluding adjacent fat and connective tissues. The software subsequently reconstructed the 3D image of the GMED with output data encompassing the muscle volume and density of the image (Fig. 1).

**Figure 2.**
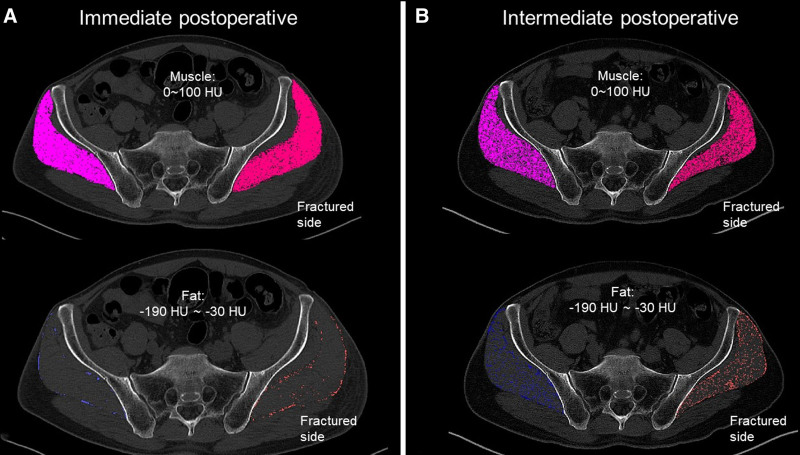
Axial computed tomography (CT) slice of the muscle (top) and adipose tissue (bottom) at the fracture (crimson/red) and uninjured sides (violet/blue) during the immediate (A) and intermediate postoperative periods (B). Over time, a reduction in the area of muscle tissue and a corresponding increase in the area of adipose tissue can be observed.

The degree of MAT changes of the GMED was assessed by calculating the mean CT density, measured in Hounsfield units (HU), as described by Goodpaster et al (−190 HU < fat < −30 HU; 0 HU < muscle < 100 HU).^[13]^ In each cross‑sectional slice, we discriminated adipose tissue and lean muscle based on these HU thresholds and calculated the lean muscle-to-adipose tissue ratio for GMED. These segmented slices were then stacked to generate a 3D reconstruction, from which the overall muscle MAT was finally calculated. The MAT ratio of the GMED muscle was represented as the ratio of the area of adipose tissue (−190 to −30 HU) against the area of muscle tissue (0−100 HU) (Fig. 2).

### 2.4. Statistical analyses

Statistical analyses were conducted using SPSS version 21.0 (IBM Corp., Armonk, NY) and SAS version 9.4 (SAS Institute, Cary, NC). Quantitative data comparing the fractured and uninjured sides were analyzed using the Mann–Whitney *U* test in SPSS. The volume change in the time group after surgery, compared with the preoperative muscle volume on the fractured and uninjured sides, was analyzed using a general linear model with repeated measures using SAS. Statistical significance was set at *P* < .05.

## 3. Results

The mean age of the 33 patients was 51 years (range, 25–62 years). Fractures were classified using the Garden and Pauwels system (Table 2). The surgical implants used were cannulated screws (3 cases), angled blade plates (2 cases), dynamic hip screws (DePuy Synthes, Zuchwil, Switzerland) with anti-rotation screws (14 cases), and femoral neck systems (DePuy Synthes, Zuchwil, Switzerland) (14 cases). Fracture reduction was performed via closed and open reduction in 21 and 12 patients, respectively. Bone union was achieved in 26 patients (78.8%), with an average union time of 5.5 months (range, 3–12 months) and femoral shortening of 3 mm (range, 0–15 mm), with 23 patients (69.7%) showing no shortening or shortening of ≤5 mm.

**Table 2:** Demographic data.

Age (yr)	51.38 ± 13.5	Co-morbidities	
Sex: male/female	13/20	Hypertension	4
Laterality: right/left	14/19	Diabetes	2
Fracture classification		Coronary disease	1
Garden classification (I/II/III/IV)	0/9/20/4	Asthma	1
Pauwel classification (I/II/II)	3/8/22	Liver cirrhosis	1
Reduction technique		Depressive disorders	2
Closed reduction	21	Epilepsy	1
Open reduction (via Smith–Peterson approach)	12		
Fixation method			
Cannulated screws	3		
Angled blade plate	2		
DHS + anti-rotation screw	14		
FNS	14		

Values are presented as mean ± standard deviation or n.

DHS = dynamic hip screw, FNS = femoral neck systems.

Preoperative and immediate postoperative GMED volumes were not significantly different between the fractured and uninjured sides. However, significant reductions in the muscle volume were observed on the fractured side during the early, intermediate, and late postoperative periods (Table 3). The MAT ratio showed no significant difference between the sides in the preoperative and immediate postoperative periods, with a slight difference in the early postoperative period. A significant increase in the fractured side ratio was observed during the intermediate and late postoperative periods (Table 3).

**Table 3:** Volume and MAT ratio of the GMED in each time group.

Group	Fractured side	Uninjured side	*P*-value
GMED volume (cm^3^)			
Preoperative	234.17 ± 65.80	228.38 ± 55.36	.196
Immediate postoperative	217.72 ± 61.17	219.32 ± 52.93	.715
Early postoperative	187.82 ± 58.41	209.20 ± 54.21	.001*
Intermediate postoperative	198.90 ± 45.30	226.49 ± 50.99	<.001*
Late postoperative	202.23 ± 35.27	217.01 ± 43.94	.046*
MAT ratio of the gluteal muscles (%)			
Preoperative	7.06 ± 5.45	6.46 ± 4.87	.564
Immediate postoperative	8.57 ± 7.04	7.85 ± 6.43	.783
Early postoperative	9.39 ± 5.77	6.39 ± 4.79	.074
Intermediate postoperative	14.45 ± 13.50	7.44 ± 6.63	.007*
Late postoperative	8.76 ± 6.46	5.53 ± 4.67	.038*

Values are presented as mean ± standard deviation.

GMED = gluteus medius, MAT = muscle-to-adipose tissue ratio.

* Statistical significance at *P* < .05.

**Figure 3.**
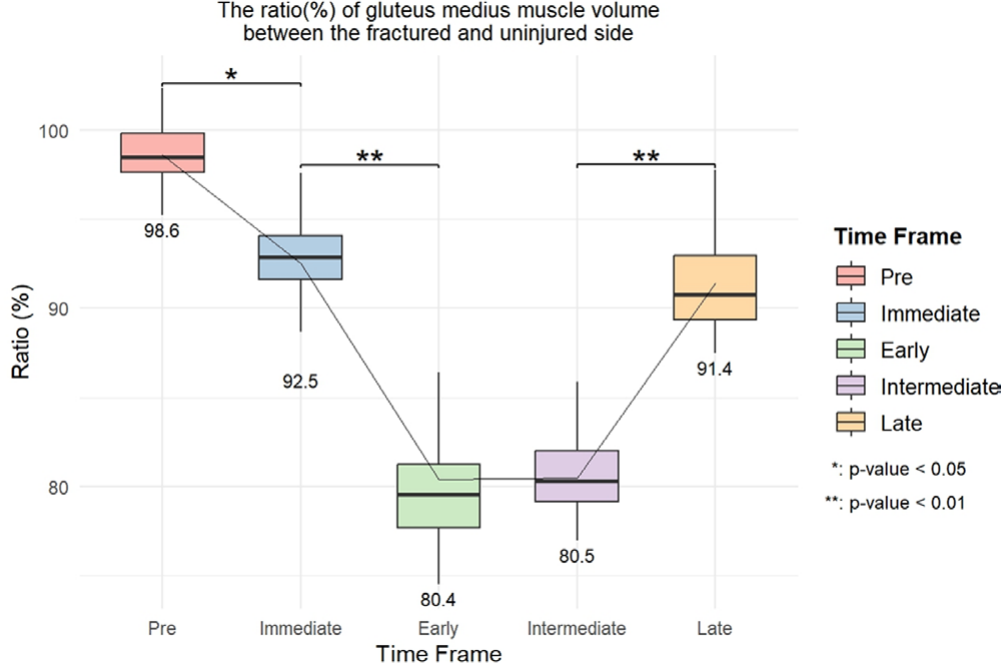
Ratio of the gluteus medius muscle volume between the fractured and uninjured sides.

**Figure 4.**
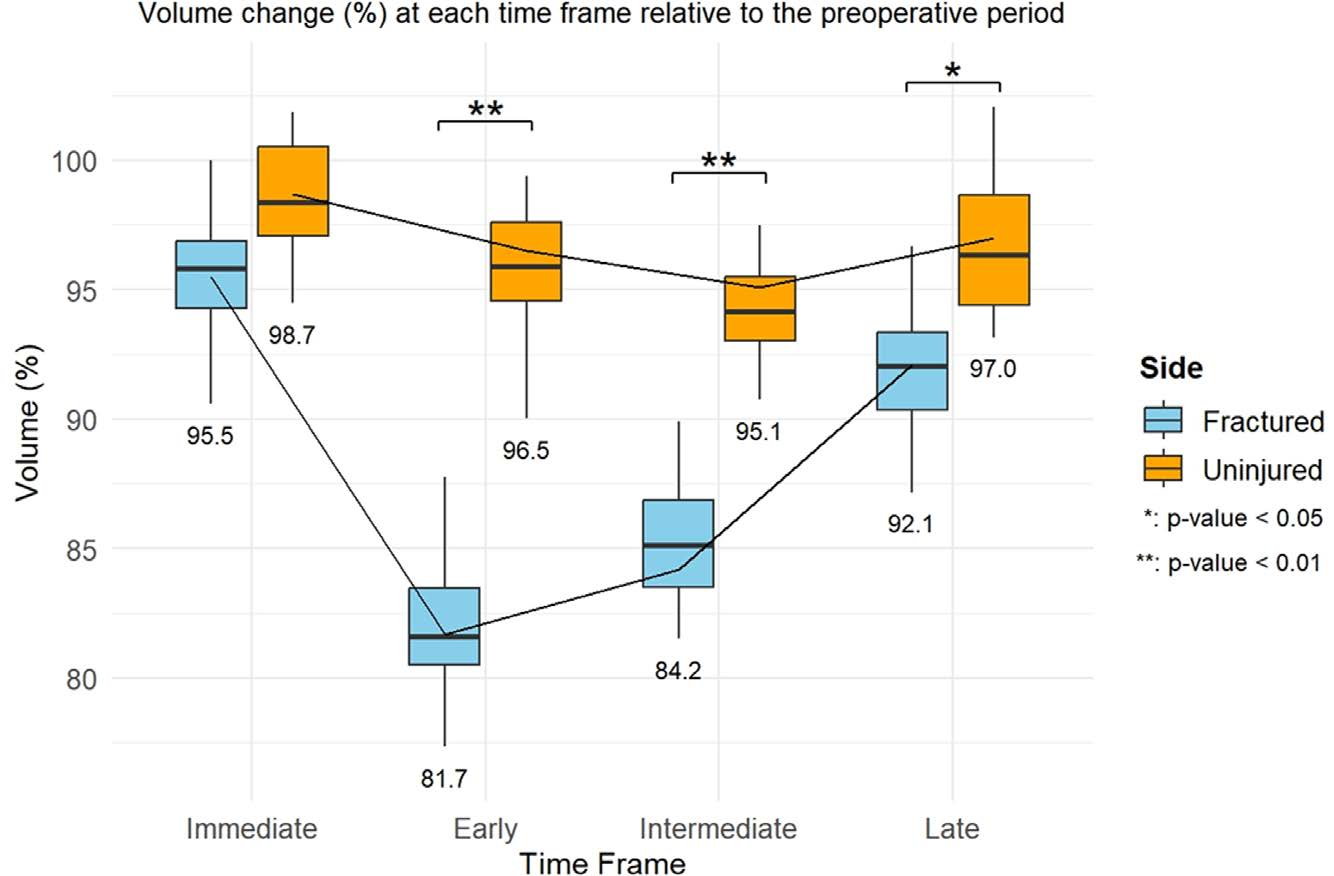
Volume change (%) in the uninjured (top) and fractured sides (bottom) determined through a comparison of the pre- and postoperative muscle volumes between the preoperative group and the immediate, early, intermediate, and late postoperative groups.

**Figure 5.**
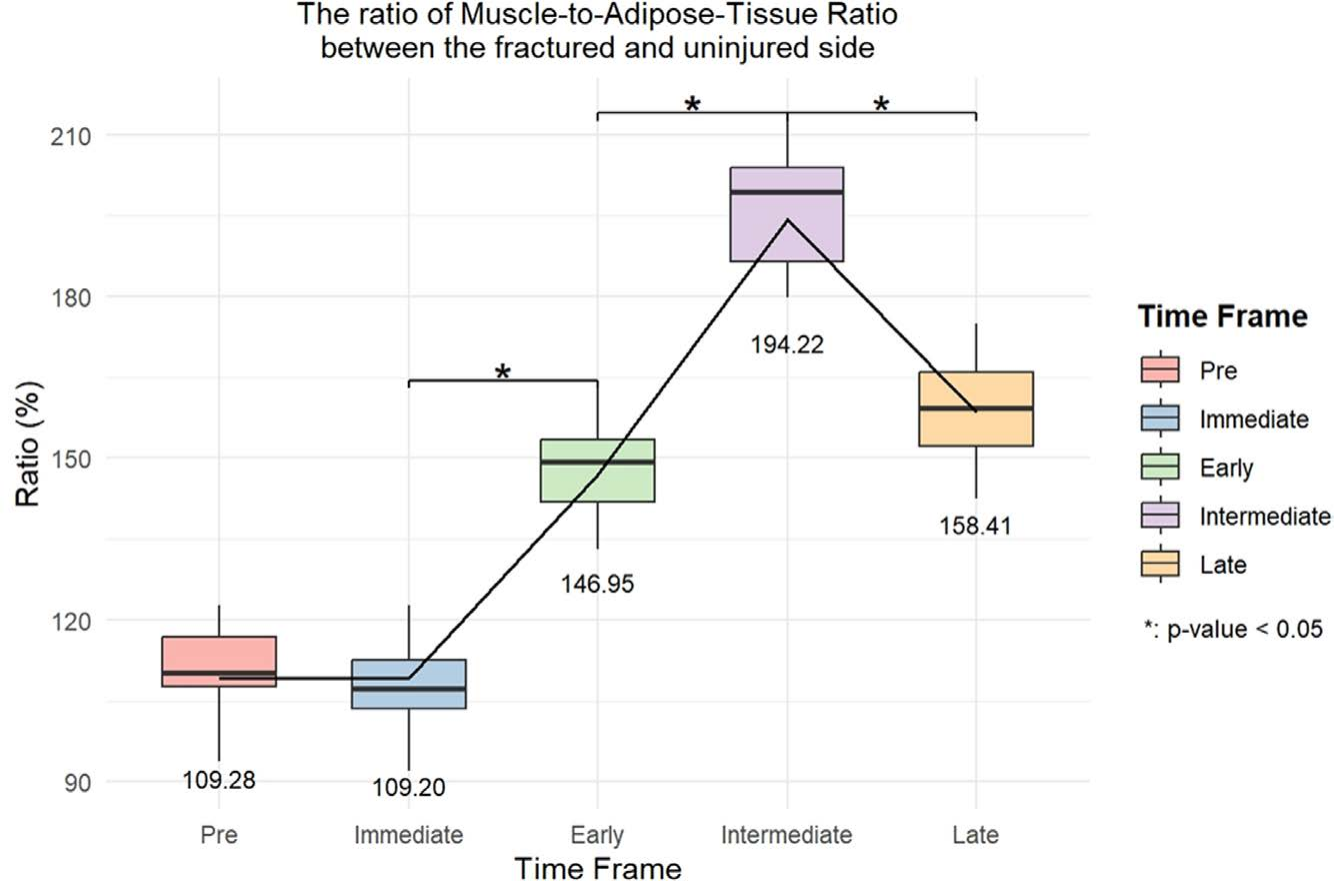
Ratio of the muscle-to-adipose tissue ratio between the fractured and uninjured sides.

Using the uninjured side as reference, the GMED volume was 98.6% preoperatively, but decreased to 92.5% immediately postoperatively (Fig. 3). The volume decreased further during the early (80.4%) and intermediate (80.5%) postoperative periods, with partial recovery (91.4%) in the late period. The general linear model revealed a minimal volume decline on the uninjured side, reaching 95.1% in the intermediate postoperative period. In contrast, the fractured side showed significant declines of 95.5% (immediate), 81.7% (early), 84.2% (intermediate), and 92.1% (late) (Fig. 4). The MAT ratio on the fractured side was 1.5 times higher than that on the uninjured side in the early postoperative period, increasing to 2 times in the intermediate period, and decreasing to 1.6 times in the late period (Fig. 5).

Among the 33 patients, 4 underwent total hip arthroplasty for osteonecrosis and 3 required revision surgery for nonunion. No significant correlation was found among avascular necrosis, union status, femoral shortening, reduction method, surgical instrument, or changes in GMED volume.

## 4. Discussion

This study evaluated the changes in muscle volume and intramuscular adipose tissue following osteosynthesis, and an important finding is that the GMED volume on the fractured side decreased by up to 20% and remained reduced by 10% even 1 year after femoral neck fracture surgery, with a 1.6 times higher MAT ratio than that on the uninjured side. The most significant volume difference between the fractured and uninjured sides occurred in the early postoperative period, with a reduction of −19.6%, which remained similar at −19.5% in the intermediate period. Muscle atrophy can be triggered by mechanical unloading, postoperative immobilization, direct muscle injury, or aging.^[14]^ Initiating hip range of motion exercises immediately after surgery and commencing partial weight-bearing crutch gait 1 month post-surgery did not prevent muscle wasting, which persisted up to 1 year post-surgery. Consistent with previous studies, the decrease in the CSA recovered less rapidly. Rasch et al^[15]^ reported significant decreases in both CSA and radiological density of the hip muscles even 2 years after total hip arthroplasty, and Mikkola et al^[16]^ found that knee extension strength and calf muscle CSA were reduced in the fractured leg of older adults 3.5 years after hip fracture. The fact that the muscle volume of the fractured side showed an increasing trend even 1 year after surgery underscores the necessity of a long-term rehabilitation program.

In addition to the comparison between the fractured and uninjured sides, statistical analysis using repeated data within each side showed that although the volume on the uninjured side decreased, the change was not significant. In contrast, the muscle volume on the fractured side sharply decreased to 81.7% in the early postoperative period and to 84.2% in the intermediate period, indicating a more dramatic reduction.

These results suggest that in healthy adults, due to abductor weakness on the injured side following surgical treatment of femoral neck fractures, difficulties with the Trendelenburg gait or side-to-side walking can persist for up to 1 year. This could be an indicator of a need to modulate the postoperative rehabilitation protocol. Additionally, although postoperative rehabilitation is necessary for both sides, specialized muscle-strengthening exercises are particularly important to prevent side-to-side differences.

Although the muscle volume on the fractured side decreased by 20%, the MAT ratio increased from the early to the intermediate period, showing a 2-fold difference before decreasing to a 1.6-fold difference in the late period. Given that muscle volume and radiological density indirectly reflect muscle strength, an increase in the MAT ratio suggests more significant qualitative than quantitative changes.^[17]^

Alterations in the musculature around the hip following surgical intervention of the proximal femur have been documented in prior literatures.^[11,18–20]^ The commonality among these studies is the measurement of muscle changes through CSA analysis. Although CSA is closely linked to muscle volume and radiological density, which indirectly reflect muscle strength, it may be less precise than directly measuring muscle volume.^[17]^ Additionally, pre-injury CSA measurements may be less accurate for comparison with post-surgery CSA because of challenges in achieving leg symmetry during imaging, as patients often cannot internally rotate both legs due to pain.^[18]^ To address these limitations, this study used CT-based 3D analysis.

Previous studies suggested that a decrease in abductor power and volume makes patients more vulnerable to femoral neck fractures.^[10,21–23]^ Moreover, in patients who have already experienced such fractures, there is an increased risk of peri-implant fractures or new fractures on the uninjured side.^[24]^ However, in our study, no new fractures were observed during the follow-up. This discrepancy is likely because our study cohort comprised relatively younger patients, unlike previous studies that focused on older patients with hip fractures. However, if such differences persist in the gluteus muscle, they may affect the risk of secondary fractures over time.

Various methods have been used for the fixation of femoral neck fractures, and their impact on the GMED may vary depending on the surgical technique and implant.^[25]^ It was hypothesized that open reduction would have a greater impact on the muscles than closed reduction. Additionally, femoral neck systems may involve less muscle dissection, whereas dynamic hip screws may cause more muscle damage. Although our study did not reveal significant differences in the GMED volume or MAT ratio based on the surgical technique or implant, the lack of significance may be attributed to the small sample size.

This study has certain limitations. First, the sample size is relatively small. Second, not all patients had CT scan data at every time point, leading to measurement discrepancies across time periods. Third, because functional scores and muscle strength were not assessed, comparisons between clinical outcomes and radiological findings could not be made. Fourth, the examiners were not blinded to the operated side, which introduced a potential bias. Despite these limitations, our findings offer preliminary confirmation of gluteus medius volume loss and fat infiltration following femoral neck fractures. These results may serve as a foundational reference for future investigations integrating surgical technique, gait analysis, and long-term rehabilitation outcomes.

## 5. Conclusion

Our study provides insights into the qualitative and quantitative changes in the GMED during the postoperative follow-up period after osteosynthesis for femoral neck fractures. Even young and healthy patients can experience GMED muscle imbalance after femoral neck fracture surgery, and this imbalance persists even after 1 year. Therefore, surgeons should carefully monitor the long-term postoperative functional status of patients in the long term and provide appropriate rehabilitation programs.

## Author contributions

**Conceptualization:** Jeong Seok Choi, Jae-Woo Cho.

**Data curation:** SungJin Ahn, Wonseok Choi.

**Formal analysis:** Seonghyun Kang, Yun Ki Ryu.

**Investigation:** Jeong Seok Choi, Jae-Woo Cho.

**Methodology:** Jeong Seok Choi, William T. Kent, Jong-Keon Oh.

**Project administration:** Jeong Seok Choi, Jae-Woo Cho.

**Writing – original draft:** Jeong Seok Choi.

**Writing – review & editing:** William T. Kent, Jong-Keon Oh, Jae-Woo Cho.
